# A CRISPR/Cas9-generated cdc-7 loss of function mutation does not cause temperature-dependent fertility defects

**DOI:** 10.17912/micropub.biology.000085

**Published:** 2019-01-03

**Authors:** Heather Currey, Nicole Liachko

**Affiliations:** 1 Geriatrics Research Education and Clinical Center, Veterans Affairs Puget Sound Health Care System, Seattle, WA 98108, USA; 2 Division of Gerontology and Geriatric Medicine, Department of Medicine, University of Washington, Seattle, WA 98104, USA

**Figure 1:**
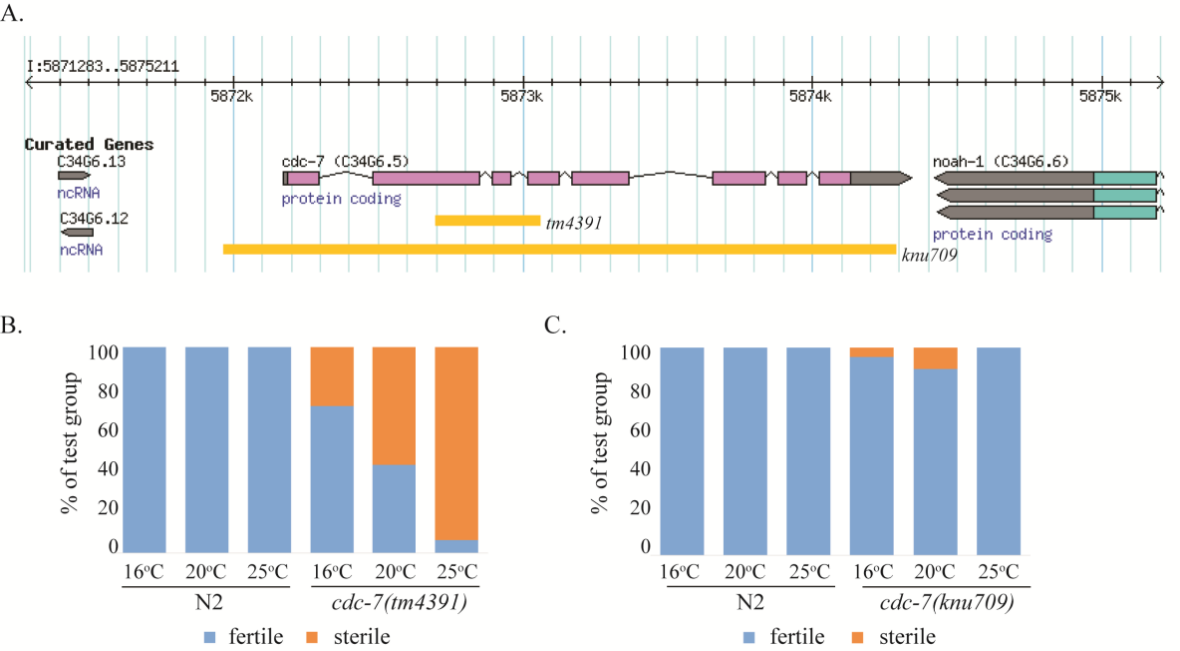
*cdc-7*(*tm4391*) but not *cdc-7*(*knu709*) is temperature-sensitive sterile. (**A**) Schematic of *cdc-7* gene locus and location of *tm4391* and *knu709* deletions. *knu709* deletes 2,284 nucleotides, beginning 163 nucleotides upstream of the *cdc-7* ATG start and ending 136 nucleotides downstream of the *cdc-7* TAA stop, within the *cdc-7* 3’UTR. This deletion does not impact upstream or downstream genes. **(B)** *cdc-7(tm4391)* worms are temperature sensitive sterile, with higher temperatures being progressively worse for fecundity. At 25^o^C, 90% of individual worms tested were sterile (Total n= 48. Number of plates with progeny = 3, number without progeny = 45). There was an intermediate effect at 20^o^C with 56% sterility (Total n= 49. Number of plates with progeny = 21, number without progeny = 28). *cdc-7(tm4391)* was most fertile when kept at 16^o^C, with only 30% sterility (Total n= 49. Number of plates with progeny = 35, number without progeny = 14). N2 controls had no observed sterility at any temperatures (for 25^o^C, 20^o^C and 16^o^C respectively, total n= 50, 50, 50. Number of plates with progeny = 50, 50, 50). **(C)** *cdc-7(knu709)* were substantially less likely to be infertile and infertility did not correspond to temperature (for 25^o^C, 20^o^C and 16^o^C respectively, percent sterile = 0%, 10%, 4%. Total n= 50, 49, 45. Number of plates with progeny = 48, 44, 43. Number of plates without progeny = 0, 5, 2).

## Description

CDC7 regulates both DNA replication initiation and checkpoint-regulated progression of the cell cycle during the G1/S phase, contributes to DNA recombination and damage repair, and is an essential gene in many species (Yamada *et al.* 2014). In *C. elegans*, there has been a single characterized deletion allele available, *tm4391*, impacting the 8-exon coding sequence of *cdc-7*. *tm4391* is missing 315 bp including part of exon 2, all of exon 3, and part of exon 4, and resulting in a downstream frame shift. *cdc-7*(*tm4391*) worms are temperature sensitive sterile, with worse fertility at higher temperatures (Figure 1B). We generated a novel deletion allele *knu709*, which is an approximately 2200 bp deletion spanning 150 bp upstream of exon 1 to about 150 bp after exon 8 and removing the entire *cdc-7*coding sequence. We tested *knu709* for temperature sensitive sterility. Surprisingly, this strain is not temperature sensitive sterile (Figure 1C), indicating that the fertility defects of *tm4391* may be due to either a closely linked mutation in another gene or an unexpected gain-of-function or partial loss-of-function phenotype of *cdc-7*(*tm4391*) not recapitulated by a true loss-of-function mutation.

## Reagents

Strains: 

CK609 *cdc-7(tm4391)* I *–* outcrossed 4x to N2.

NLS1 *cdc-7(knu709)* I *–* outcrossed 3x to N2. Strain will be sent to CGC.

## Methods

Genotyping:

Oligos used for genotyping *tm4391:* Mix all 3 primers in a single reaction. Expect sizes 757bp and 270bp WT, 400bp mutant.

NL93 (F): tcagtgcaacatgcagaaca

NL94 (R): tgacacaaaccaatcccaaa

NL187 (internal deletion): ATGCGACAGCATAAAGCAAA

*Oligos used for genotyping knu709:* Mix all 3 primers in a single reaction. Expect sizes 2848bp and 823bp WT, 565bp mutant.

NL429 (F): cccgtatcacacactcatcg

NL430 (R): attgctaaaacccgcagaaa

NL431 (internal deletion): ggaaacgtaccctcgcctat

Fertility Assays

Synchronized populations of C. *elegans* were established by treating with alkaline hypochlorite solution (bleaching) (Porta-de-la-Riva *et al.* 2012). Eggs were grown at 16^o^C to L4 stage at which point worms were singled onto 60mm NGM plates seeded with OP50. Plates with singled worms were then divided into 3 groups of approximately 50 per strain. Groups were transferred to the assay temperature: either 25^o^C, 20^o^C or 16^o^C. After 1 week, plates were scored for the presence of progeny. The absence of progeny was scored as sterile.

CRISPR/Cas9

COP1803 *cdc-7(knu709)* was generated by Knudra Transgenics/ NemaMetrix (Eugene, OR), and the deletion end-points confirmed by sequencing. 3-frame stop insertion sequence at breakpoints of deletion: TAAATAAATAAACTCGAG.
